# Character Strengths as Manifestations of Spiritual Life: Realizing the Non-Dual From the Dual

**DOI:** 10.3389/fpsyg.2020.00960

**Published:** 2020-05-26

**Authors:** Hadassah Littman-Ovadia, Amnon David

**Affiliations:** ^1^Department of Behavioral Sciences and Psychology, Ariel University, Ariel, Israel; ^2^No Academic Affiliation, Tel Aviv, Israel

**Keywords:** virtues, character strengths, VIA, spirituality, non-duality, duality, paradox, transcendence

## Abstract

There is a noticeable increase in interest in the study of spirituality within the context of positive psychology. A review of the literature shows several parallels between dimensions of spirituality as explored within psychology of religion and spirituality and those of the VIA model of character strengths (CSs) as developed in positive psychology. However, coming from the domain of psychology rather than theology, these studies do not go deeply into the paradoxes that exist at the heart of various traditions regarding the nature of the spiritual or non-dual. Moreover, these studies lack a more comprehensive view of the nature of CSs and virtues. Our suggestion is to expand CS science to a wider context, extend the perspective from the individual to the transcendent, and understand the actualization of the capacity of CSs to be pathways to spiritual life. We argue that the actualization of all CSs allows for microcosms of a realization of unity. We believe that framing VIA’s CSs as a classification of the positive human spirit, and therefore rightfully placing it in the domain of human spirituality, holds great potential for both domains. We start by considering common basic assumptions emerging from various spiritual traditions and continue with a suggestion that CSs be seen as various pathways from duality to non-duality and by illustrating ways in which spirituality can be understood and practiced by the use of CSs.

*Even if there is only one possible unified theory*, *it is just a set of rules and equations. What is it that breathes fire into the equations and makes a universe for them to describe? Why does the universe go to all the bother of existing?*
–Stephen Hawking, A Brief History of Time

## Introduction

The increase in the number of studies on spirituality within the context of positive psychology is not surprising given the fact that both fields focus on what is needed for individuals to be good and to live well. Spirituality, as explored within the psychology of religion and spirituality, and the VIA model of character strengths (CSs) and virtues, as developed by positive psychology, both highlight the moral importance of cultivating dispositions such as generosity, courage, humility, love, and honesty. The question of human goodness is also central in applied virtue theory, which has recently become a vibrant area in the field of applied ethics, and indeed virtue philosophers emphasize the moral importance of character traits (Axtell and Olson, 2012).

However, these studies do not address the wider context of spirituality in general or of transcendence in particular. Moreover, these studies lack a wide perspective on the spiritual nature of CSs and virtues. We argue that actualization of CSs allows for microcosms of a realization of unity/wholeness or, in more spiritual terms, touching the paradox of realizing the non-dual from the dual. Thus, this paper proposes a new perspective for understanding the relationship between elements of spirituality and CSs. We believe that framing VIA’s CSs and virtues as a classification of the positive human spirit, as well as connecting it with the domain of human spirituality, holds great theoretical and practical potential for both domains. We start out by considering common basic assumptions emerging from the study of various spiritual traditions, we continue with a suggestion that the CSs of the VIA classification should be seen as both manifestation and realization of spirituality, and we end with a discussion of the implications of the proposed perspective.

## Spiritual Common Aspects

The term spirituality means different things to different people. It originates from the Latin word spiritus, meaning vapor, breath, air, or wind. Webster’s dictionary defines spirituality as: “relating to, consisting of or affecting the spirit; relating to sacred matters; concerned with religious values; related to, or joint in spirit” (Spirituality, 2019). Mitroff and Denton (1999) defined spirituality as the desire to find one’s ultimate purpose in life and to live accordingly. Another scientific definition of spirituality is “search for the sacred,” where the sacred is characterized by three qualities: transcendence (a sense of being in touch with something beyond ordinary experience), boundlessness (lacking the boundaries and limits of ordinary life), and ultimacy (the quality of being “basic and elemental” or deeply true) (Pargament, 2007, p. 39). However, a review of the literature determined that there is no single agreed-upon definition of the term spirituality among those who conduct research in this field.

Moreover, disagreement exists not only on the definition of spirituality but also regarding whether there is a common denominator among the different spiritual traditions. Perennialism (or perennial wisdom) is a perspective on spirituality that views all the world’s spiritual traditions as sharing a single, metaphysical truth or origin, one universal reality that is experienced through multiple paths (Tyson, 2012). Constructivism is a perspective that is closely related to pluralism, relativism, and subjectivism and asserts that there is no objective reality or innate experience that is independent of mental and cultural constructs. Thus, it is the specific spiritual tradition in question that determines what “reality” is as well as how to achieve spiritual union with it (Tyson, 2012).

In the perennialism vs. contextualism debate, we adopt Smith’s (1987) suggestion that despite these two approaches often being seen as opposed alternatives, both are actually shaped by a set of complementary epistemological assumptions. This also suggests that different theological classifications (such as monism and monotheism) can be seen as complementary views of the ultimate, which means that both a unique and personal experience of a benevolent God by a Christian mystic and a metaphysical recognition of a Zen master can be seen as different aspects of divine presence.

The landscape of spirituality is immense, so we will focus on two basic claims. The first being the existence and primacy of an ineffable knowledge, a unity that escapes all attempts at being defined or categorized via any concepts or abstractions. This non-dual knowledge is at the core of different and diverse mystical teachings, is prior to concepts, and is therefore resistant to any attempt at capturing its essence using concepts or definitions (Wildman, 2006).

The second claim is the existence of an inevitably paradoxical connection with this knowledge, which stems from the fact that while the essence of existence is non-dual, experience can only occur in what appears to be a dualistic world of objects and subjects. While explaining the non-dual is impossible (as any concept implies a context in which it can be understood, thereby necessitating duality), through the ages, varied traditions developed methods to point to it (e.g., Farley, 2011; Zaki, 2019). This attempt to touch the non-dual is the transcendence element mentioned in most definitions of spirituality and shown to be spirituality’s central aspect in a review of 22 papers (McCaroll et al., 2005). An integrative research review of 20 studies published between 2007 and 2017 showing the health benefits of transcendence also mentions it as the central aspect of spirituality (Counted et al., 2018).

Duality stems from a seeming division between a belief in a reality “out there” experienced by a someone “in here.” It enables concepts, thoughts, and, consequently, – science (Servajean, 2008). In an attempt to avoid dualism, which would imply dimensions beyond the grasp of science, a philosophy of physicalism or materialism that rejects any spiritual aspects was adopted by many scientists (see discussion on this topic in Gebelein, 2013). This philosophy has proven useful, but while aimed at achieving a unified, consistent understanding of reality, it ironically perpetuates the very dualism it tries to avoid because unlike a unified reality prior to concept formation, material objects are always conceptual models built from experiences of a necessarily separate subject.

Despite rivalries in their interpretation, spirituality and mysticism have influenced prominent developers of modern physics (Marin, 2009). *…“it is the hallmark of any deep truth that its negation is also a deep truth”* (Delbrück, 1986, p. 167). This quote by Niels Bohr introduces a recurring aspect of spirituality: the inherent paradoxes that arise when attempting to make sense of spiritual insights.

These paradoxes do not imply an error in reasoning but rather a limitation of reasoning when attempting to grasp that which exists even prior to reason, or, in spiritual terms, when attempting to comprehend, using dualistic tools, non-dual truth, a truth that already exists before any personal experience, thought or memory and yet something we all have direct and intimate access to. Disentangling from a limited perspective of self and realizing a deeper dimension of consciousness or the nature of truth is the essence of spirituality, and observing spiritual exemplars (Scarlett, 2012; Vos, 2018) shows a strong association between this realization and CSs and virtues. We propose that the VIA model of CSs and virtues is an appropriate framework for understanding, practicing, and experiencing the paradoxical relationship between the *personal* and the *transcendent*.

## The VIA Character Strengths and Virtues

Positive psychology has changed the face of the field over the last two decades, setting a mission of creating a world where psychologists do not simply treat mental illness but rather help individuals to improve and live a full life (Park et al., 2004). A core endeavor in the field of positive psychology has been the identification of positive individual traits (Seligman and Csikszentmihalyi, 2000). The first substantial effort to achieve this goal took the form of a manual defining good character and describing the right and the positive about individuals – the CSs and Virtues (CSV) classification of strengths (Peterson and Seligman, 2004).

The development of the CSV was achieved through an extensive 3-year research project examining the philosophical and religious traditions of China (Confucianism and Taoism) and South Asia (Buddhism and Hinduism) and those of the West and Ancient near East (Ancient Greek philosophy, Judaism, Christianity, and Islam). In order to identify CSs from the gathered material, a number of criteria were established, such as traits having to be morally valued regardless of beneficial outcomes their use may lead to and being non-diminishing of other individuals.

This effort resulted in the classification of 24 CSs that reflect durable positive individual tendencies for feeling, behaving, and thinking, categorized across six universally valued virtues. CSs are viewed as the “psychological ingredients – processes or mechanisms – that define virtues” (Peterson and Seligman, 2004, p. 13) and provide distinguishable ways to display each virtue. The virtue of wisdom can be achieved through creativity, curiosity, love of learning, critical thinking, and perspective. Courage is composed of the CSs of bravery, honesty, persistence, and zest. Humanity includes kindness, love, and social intelligence. Justice envelopes teamwork, fairness, and leadership. The path to temperance is paved by forgiveness, modesty, prudence, and self-regulation. Transcendence includes spirituality, appreciation of beauty, gratitude, hope, and humor.

## VIA’s CSs: to Spirituality and Back

As the VIA framework offers a comprehensive model that is not based on or limited to a single spiritual tradition, and as hundreds of studies consistently provide support for the beneficial nature of VIA CSs and their contribution to a wide range of desirable outcomes (Littman-Ovadia and Niemiec, 2016), we suggest that VIA’s CSs be framed as a classification of the positive human spirit. From a top-down perspective, we suggest that CSs be viewed as the various ways in which non-duality is manifested in duality (human conduct), as implied by the VIA founders’ belief that strength and virtue are an essential aspect of the transcendent that exists within each human being:

*According to the Judeo-Christian account of the genesis of human life*, *the physical entity that was the first human became fully alive only after God breathed “the breath of life” into him. Through that*… *act of intimacy he imparted an essential*, *enlivening*, *divine*, *and sacred aspect of himself into each human being. This divine breath of life*… *is believed to be the source of human strength and virtue. the source of the capacity for creativity*… *the capacity for love*, *intimacy*, *harmony*, *growth*, *compassion*, *goodness*, *and optimism* (Peterson and Seligman, 2004, p. 602).

Viktor Frankl’s ideas about spirituality and virtues, and Wong’s (2014) extensions of this work, seem highly consistent with the top-down perspective espoused here. According to Frankl (1985), self-transcendence is the ultimate end in life and the main purpose of human existence, as self-transcendence involves a purposeful life that is dedicated to loving others or serving a cause greater than one’s self. The pursuit and attainment of transcendental values leads to the deepest satisfaction because it satisfies the deepest yearning of our spiritual nature. According to Wong (2014), this is why Frankl has argued that we become fully functioning human beings only when we lose ourselves in self-transcendental pursuit.

From a bottom-up perspective, we suggest that CSs be viewed as various pathways from duality to non-duality. The most obvious of these is the *transcendence* virtue, characterized by the common theme of allowing individuals to forge connections with the larger universe, thereby providing meaning to their lives. *Spirituality* refers to a belief in and commitment to the non-materialistic aspects of life and having coherent beliefs about the higher or ultimate purpose and meaning of the universe and of the individual’s life within it. People with this strength prioritize moral values and have an interest in the pursuit of goodness. *Appreciation of beauty and excellence* describes noticing and appreciating beauty and excellence in different domains of life. This CS is part of the virtue of transcendence because it connects those who possess it to something larger than themselves, whether it be beautiful art or music, a skilled athletic performance, the majesty of nature, or the moral brilliance of other people. *Gratitude* describes having a sense of thankfulness in response to a tangible or abstract gift provided by a specific or non-specific other person or by nature or the universe. *Hope* describes thinking about the future, expecting the coming of desired events and positive outcomes, feeling confident that these might well ensue given appropriate efforts, and finally, making these efforts. Hope represents having an attitude that is turned toward the goodness that the future might hold, be it specific positive outcomes or broader desires. *Humor* describes a tendency to laugh and gently tease, to make others smile, to see the light side of life situations, and to make the human condition more bearable by drawing attention to its contradictions or by building social bonds. As humor is rarely mentioned explicitly by philosophers and theologians, Peterson and Seligman (2004) classified it as a value-added strength – most praiseworthy when coupled with another strength (Peterson and Seligman, 2004).

But, as mentioned above, not only the *transcendence* virtue is essential for realization. As can be seen in spiritual exemplars, a common characteristic of those who live the depth of this truth is conduct that is abundant in love, kindness, humility, patience, compassion, equanimity, joy, emotional stability, critical thinking and clarity, spontaneity, leadership, persistence, and a grounded inner strength that enables them to face any adversity. In other words, they exhibit a powerful manifestation and integration of all CSs and virtues (Scarlett, 2012; Vos, 2018).

Character strengths and virtues can serve as pathways to transcending the deterministic game. While psychology can and does study various limitations of free choice or how its perceived existence influences behavior, it cannot answer age-old metaphysical questions on its meaning or validity (Baumeister, 2008). Spirituality suggests that the confusion regarding personal freedom is derived from confusion regarding a separate personal self, and the journey into higher virtues is one of exercising choice by transcending the perceived self in a given situation. The following analogy illustrates a Jewish perspective of the process.

*“When two armies are locked in battle*, *fighting takes place only at the battlefront. If one side gains a victory at the front and pushes the enemy back*, *the position of the battlefront will have changed. The situation is very similar with regard to [moral choice]. With each good [choice] successfully carried out*, *the person rises higher in spiritual level; that is*, *things that were previously in the line of battle are now in the area controlled by the [good inclination] and actions done in that area can be undertaken without struggle and without [choice]”* (Dessler, 1978, p. 52–54).

As choice always exists for a limited self, paradoxically, the ultimate choice is to have no choice. The journey of spirituality can thus be viewed as a movement from vice (ignorance, lack of choice to act virtuously due to a low level of consciousness) to virtue (enlightenment, lack of choice to succumb to vices due to a higher level of consciousness). Growing in virtue can be achieved by the act of dissolving rigid concepts through the transcendence that usually follows deep introspection rather than from following preconceived external laws or notions of morality (Yan, 2009; Snow, 2016). The source of virtue is at the place where separation between the internal and external dissolves. When viewed from the perspective of separation, it is manifested as the correct application of CSs in the dualistic world that we are all familiar with. In practice, the process of acquiring virtue and spirituality is one that combines growing our humanity from the outside-in as well as from the inside-out (Vos, 2018) until the point when the very separation of inside and outside is seen as the illusion it is, although it is a very necessary illusion that is required for celebrating life.

Finally, we argue that CSs allow us to approach all spiritual components and paradoxes: bridging opposites and including the entire spectrum of experience (e.g., perspective), connectedness/wholeness (e.g., kindness), being able to see beyond cause and effect (e.g., love), allowing identification of the right action, “the next obvious step” (e.g., social intelligence), and seeing through the separate self (e.g., humility). Additional examples, in the form of the non-dual manifestations of the six virtues, are presented in Table 1.

**TABLE 1:** Non-dual manifestations of virtues.

**Virtues**	**From vice to virtue: Non-dual manifestations**
Wisdom	• Shifting from a self-focused, defensive orientation, toward a greater view that facilitates learning and growth (Crocker, 2008; Crocker et al., 2017)
	• Practical wisdom serves as a steppingstone to transcendent wisdom – worldly perfection that leads to divine enlightenment. This virtue includes understanding what is meaningful and lasting and having insight into transcendent ends rather than practical means (Peterson and Seligman, 2004)
Courage	• Shifting from a defensive and self-threatening orientation regarding failures and setbacks toward accepting responsibility and improving one’s self abilities (Crocker, 2008; Crocker et al., 2017)
	• Achievement of a healthy intimacy with Nothing-Infinite Eternal, fearlessness, allowing one to engage with what causes fear (Barnesmoore and Fisher, 2019)
Humanity	• Moving from a self-focus, which may facilitate loneliness, toward focusing on others, thereby building relationships and closeness (Crocker, 2008; Crocker et al., 2017)
	• Includes strengths that often represent self-transcendent emotions, often encouraging individuals to put aside their own needs and desires in favor of someone else’s (Stellar et al., 2017)
Justice	• Replacing the conflict and competition that may be fostered by a self-focus with collaborative and supportive relationships, fostered by a larger view (Crocker, 2008; Crocker et al., 2017)
	• Includes strengths that value social bonds, building and sustaining relationships in reflection of a self-transcendent, rather than a self-enhancing, orientation (Peterson and Seligman, 2004)
Temperance	• Moving from self-centered to eco-considerate goals, reflecting a shift from obsessive to harmonious passion (Crocker, 2008; Crocker et al., 2017)
	• This virtue includes countering the natural tendency to value oneself more than others and attending to what is truly of value in all persons (Morgan, 2001)
Transcendence	• Shifting from such self-focused emotions as pride and shame, to other-focused ones, including appreciation and gratitude (Crocker, 2008; Crocker et al., 2017)
	• This virtue is thought to be most directly related to belief and commitment to the immaterial. Within it are paths to excellence, goodness, a dreamed-of future, and direct connection to troubles and contradictions by means of pleasure rather than anger or fear (Peterson and Seligman, 2004)

## Conclusion and Implications

There are numerous advantages to this spiritual perspective on the VIA, as well as to a strengths perspective on the spiritual life.

Conceptually, it opens up a new language for researching both fields, as well as providing an opportunity to bridge them. VIA and spirituality are both morally based and thus share similar aims. We believe that every CS can be seen as a gateway to realization, as every CS is a reflection of spirituality, representing the reciprocal linkage between the fields.

Practical implications include the notion that nurturing strengths can increase the degree to which individuals and collectives can realize and celebrate the unity and connectedness of all things. Building a healthier and more moral society is possible through combining inside-out with outside-in efforts. An enlightened society will be created by having enlightened individuals in it, and enlightened individuals will emerge through their discovery of their true nature. This is related to top-down, i.e., non-dual understanding, to dual manifestation. Enlightened individuals can also emerge by following the laws of an enlightened society (it also includes emulating exemplars). This is related to bottom-up realization: following, practicing, and learning (the manifested) virtues, eventually connecting to the non-dual. In practice, there is always an interplay between top-down and bottom-up, inside-out and outside-in approaches on the way to an enlightened individual and society, but ultimately the realization is that there is no real inside or outside, top or down and that all is the play of the divine.

Of course non-dual truth, like existence, is not something that can be falsified, however, the central ideas in this paper can and should indeed be subject to possible falsification, for example, finding counter-examples to spiritual maturity being conducive to behavior that manifests human virtues. Also, more empirical studies on the correlation between non-dual wisdom and virtuous behavior could further substantiate the claims.

## Author Contributions

All authors listed have made a substantial, direct and intellectual contribution to the work, and approved it for publication.

## Conflict of Interest

The authors declare that the research was conducted in the absence of any commercial or financial relationships that could be construed as a potential conflict of interest.
